# Modulation of the gut-bone axis: *Lacticaseibacillus paracasei* LC86 improves bone health via anti-inflammatory metabolic pathways in zebrafish models of osteoporosis and cartilage damage

**DOI:** 10.3389/fimmu.2025.1493560

**Published:** 2025-04-16

**Authors:** Yao Dong, Yukun Sun, Zhipeng Zhou, Zhonghui Gai, Yihui Cai, Mei Han, Kang Zou

**Affiliations:** ^1^ Germline Stem Cells and Microenvironment Lab, College of Animal Science and Technology, Nanjing Agricultural University, Nanjing, China; ^2^ Stem Cell Research and Translation Center, Nanjing Agricultural University, Nanjing, China; ^3^ Department of Research and Development, Wecare Probiotics Co., Ltd., Suzhou, China; ^4^ Food Science and Nutrition, University of Leeds, Leeds, United Kingdom; ^5^ School of Biomedical Engineering, Hubei University of Medicine, Shiyan, China; ^6^ Department of Food Quality and Safety, Shanghai Business School, Shanghai, China

**Keywords:** osteoporosis, cartilage injury, *Lacticaseibacillus paracasei*, gut-bone axis, zebrafish model

## Abstract

**Aim:**

Osteoporosis and cartilage injury are major health concerns with limited treatment options. This study investigates the therapeutic effects of *Lacticaseibacillus paracasei* LC86 (LC86) on osteoporosis and cartilage damage in a zebrafish (*Danio rerio*) model, focusing on its modulation of the gut-bone axis and its potential mechanisms for enhancing bone health.

**Methods:**

A Dexamethasone-induced zebrafish model was used to mimic osteoporosis and cartilage injury. Zebrafish were divided into control, model, and LC86 treatment groups (3×10^7^ CFU/mL). Bone and cartilage health were assessed using Alizarin red staining and fluorescence microscopy. Bone marker expression (*sp7*, *runx2a*, *bmp2a*, *bmp4*, and *col2a1a*) was quantified via qPCR. Metabolic alterations were analyzed using untargeted metabolomics, and changes in gut microbiota were examined through 16S rRNA gene sequencing.

**Results:**

LC86 treatment significantly improved bone and cartilage health, as evidenced by increased fluorescence intensity in the skull, hard bone, and cartilage (*p* < 0.01, *p* < 0.05). qPCR results showed upregulation of key bone-related genes (*sp7*, *runx2a*, *bmp2a*, *bmp4*, and *col2a1a*), indicating enhanced bone and cartilage structure. Metabolomics analysis revealed alterations in over 300 metabolites, with changes in anti-inflammatory and energy pathways. Gut microbiota analysis demonstrated an increase in beneficial bacteria and a decrease in pathogenic genera.

**Conclusions:**

LC86 significantly improved bone health, cartilage structure, and gut microbiota composition in a Dexamethasone-induced zebrafish model, supporting its potential as a therapeutic strategy for osteoporosis and cartilage injury via modulation of the gut-bone axis.

## Introduction

Osteoporosis and cartilage injury are common skeletal health issues globally, severely affecting patients’ quality of life. Osteoporosis is a systemic metabolic bone disease characterized by reduced bone density and deterioration of bone microstructure, marked by low bone mass, worsening of bone tissue microarchitecture, increased bone fragility, and a higher susceptibility to fractures (1, 2). This condition is especially prevalent among postmenopausal women and primarily arises from an imbalance where bone resorption exceeds bone formation, leading to a reduction in bone mass (2, 3). Cartilage injury refers to the destruction or degeneration of cartilage tissue, which typically covers joint surfaces and is smooth and flexible (4). Its primary functions are to reduce joint friction, absorb impact, and protect bones. Cartilage injuries can be acute, caused by trauma or physical activity, or chronic, stemming from degenerative diseases like osteoarthritis (5). Osteoporosis and cartilage injury, as chronic and long-term skeletal diseases, share many pathophysiological features (6); the microstructural changes in osteoporosis can exacerbate cartilage degeneration, while the inflammatory response from cartilage injury may also affect bone metabolism and reconstruction processes (7). Clinically, treatment strategies for these conditions often involve using bone mineralization promoters such as Alendronate (ALN), cartilage protectants like Chondroitin Sulfate (CS), and other hormonal drugs, although these treatments may cause side effects like gastrointestinal discomfort and allergic reactions (8, 9).

In recent years, the gut-bone axis has emerged as a novel approach for the prevention and treatment of bone health issues (10). The gut-bone axis refers to the interactions between gut microbiota and bone cells that can alter bone metabolism through various mechanisms, including the production of metabolic byproducts, influencing the host’s metabolic pathways, and regulating the body’s immune system (11). The gut microbiota consists of a collection of microorganisms residing in the host’s intestine that significantly impact host health. Studies suggest that modulating the gut microbiota is a safer and more effective way to improve conditions like osteoporosis and cartilage injury. Among these approaches, the role of probiotics is particularly prominent. Probiotics are defined as “live microorganisms that, when administered in adequate amounts, confer a health benefit on the host (12).” The influence of probiotics on the gut microbiota is significant in the treatment of human and animal diseases. Their considerable potential has prompted researchers to delve deeper into the study of probiotics. *Lactobacilli* and *Bifidobacteria* are two common types of probiotics that can affect bone metabolism by altering the gut microbiota (13). Some studies indicate that probiotics can influence bone metabolism through certain metabolic pathways or gene regulation, thereby improving the health of bones and cartilage. For instance, research by Plavnik I et al. has shown that probiotics can regulate bone metabolism and repair processes by modulating the gut microbiota, host metabolism, immune responses, and cartilage development (14, 15). Similarly, Britton RA et al. reported that *Lactobacillus reuteri* can increase bone mineral content and density, thereby improving osteoporosis (16). Therefore, probiotic supplements can serve as biological markers for treating bone health issues such as osteoporosis, although their underlying mechanisms require further investigation.

Zebrafish (*Danio rerio*) have become an essential model organism for studying bone development and diseases in preclinical research. The potential of these teleost fish lies in their small size, ease of care, genetic tractability, and high regenerative capacity (17, 18). Additionally, the transparency of embryos and larvae allows for detailed monitoring of osteogenic activity and osteoblast behavior using existing transgenic lines and mutations that target specific cells or tissues (17). Moreover, the feasibility of long-term *in vivo* imaging in embryos, larvae, and adult individuals sets zebrafish apart from other vertebrate models such as rodents, where live imaging can pose challenges. Importantly, zebrafish have transparent skeletal and cartilaginous structures, rapid development, and a bone remodeling mechanism similar to humans (19). Their genome contains orthologs of approximately 82% of human disease-related genes, including those affecting bone and osteocyte signaling pathways (19). Besides, zebrafish can be administered drugs in multiple ways, including dissolving the target drug directly in the water/culture medium, which is the one of the preferred methods for drug screening.

Preliminary animal studies have found that *Lacticaseibacillus paracasei* LC86 can ameliorate issues such as muscle atrophy caused by biological aging and may play a role in maintaining immune homeostasis and regulating the gut microbiota (20). However, the specific mechanisms of action of LC86 in osteoporosis and cartilage injury remain unclear, and its regulatory effects on these bone health issues have not been fully explored. Thus, this study aims to evaluate the impact of strain LC86 on bone health by establishing a zebrafish model of dexamethasone (DXMS)-induced osteoporosis and cartilage injury. We will investigate the effects of LC86 on bone-related gene expression and assess its regulatory role on the gut microbiome through untargeted metabolomics combined with 16S rRNA gene amplicon sequencing, unveiling its potential mechanisms within the gut-bone axis. This will provide new scientific insights into the application of probiotics in bone health, potentially offering novel approaches and strategies for the prevention and treatment of osteoporosis and cartilage injuries.

## Materials and methods

### Preparation of strain LC86 and sample configuration

The strain LC86 was provided by Wecare Probiotics Co., Ltd. (Suzhou, China). The strain was cultured at 37°C in De Man, Rogosa, and Sharpe (MRS) medium for 18 hours (20). Bacterial cells were collected by centrifugation at 6000 × *g* for 8 min and resuspended in sterile water to achieve a final concentration of 1×10^9^ CFU/mL.

Alendronate sodium, used as the positive control for osteoporosis treatment, was obtained in white tablet form (Batch No. X002979, Savio Industrial S.r.l.) and dissolved in ultrapure water. Chondroitin sulfate, used as the positive control drug for cartilage injury protection, was obtained in white powder form (Batch No. I2003201, Shanghai Aladdin Biochemical Technology Co., Ltd.) and dissolved in standard dilution water.

### Equipment, supplies, and reagents

Key equipment used in this study included a dissection microscope (OLYMPUS SZX7), fluorescence microscope (Nikon AZ100), CCD camera, precision electronic balance (OHAUS CP214), ultrasonic cleaner, automatic sample grinder, automatic nucleic acid extractor (Auto-Pure32A), conventional PCR thermal cycler (BIO-RAD T100), real-time quantitative PCR instrument (BIO-RAD CFX Connect), high-speed refrigerated centrifuge, UV-Vis spectrophotometer (NanoDrop 2000), and standard 6-well plates. Major reagents included dexamethasone (DXMS), dimethyl sulfoxide (DMSO), methylcellulose, alizarin red, Universal RNA Extraction Kit, FastKing cDNA First Strand Synthesis Kit, and ChamQ Universal SYBR qPCR Master Mix. All reagents were obtained from commercial suppliers and used according to the manufacturers’ protocols.

### Experimental animals

This study utilized two zebrafish strains: the wild-type AB strain and the transgenic cartilage green fluorescent (Tg (OlaSp7:nlsGFP) cy25) zebrafish. All zebrafish were maintained in custom aquaculture water at 28°C, which was prepared by adding 200 mg of instant sea salt per liter of reverse osmosis water. The conductivity was maintained between 450-550 μS/cm, with a pH range of 6.5-8.5, and water hardness between 50-100 mg/L CaCO_3_. The zebrafish were bred and provided by Hunter Biotechnology Inc. (Hangzhou, China) aquaculture center. This experiment received an animal use permit (Permit Number: SYXK (Zhe) 2022-0004) and strictly adhered to the standards of the Association for Assessment and Accreditation of Laboratory Animal Care International (AAALAC) (Certification Number: 001458). All experimental procedures were ethically reviewed and approved by the Institutional Animal Care and Use Committee (IACUC) (Review Number: IACUC-2024-8511-01).

### Three zebrafish models for multi-dimensional assessment of LC86 on bone health

**Figure 1:**
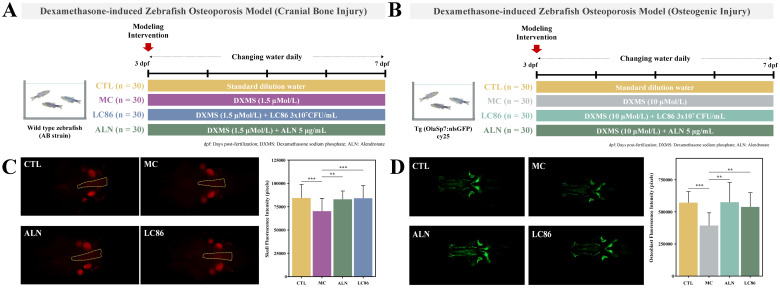
Establishment of a Dexamethasone (DXMS)-induced osteoporosis model in zebrafish and the effects of *Lacticaseibacillus paracasei* LC86 intervention. **(A)** Cranial bone injury; **(B)** Osteogenic injury; **(C)** Fluorescence intensity and quantification of zebrafish cranial bones following LC86 intervention; **(D)** Fluorescence intensity and quantification of hard bones in zebrafish after LC86 intervention. **indicates *p* < 0.01.

DXMS-induced cranial bone injury model in wild-type zebrafish (21, 22): To investigate the impact of dexamethasone (DXMS) on cranial bone mineralization, a cranial bone injury model was established using 3 days post-fertilization (dpf) wild-type AB strain zebrafish. Zebrafish were randomly assigned to four groups: control (CTL), model control (MC), positive control (ALN), and LC86 treatment group. Thirty fish were maintained in 20 mL of culture water per beaker. Except for the CTL group, zebrafish in the MC, ALN, and LC86 groups were exposed to 1.5 μM DXMS for 4 consecutive days to induce cranial bone injury. The ALN group received 5.00 μg/mL of alendronate sodium, while the LC86 group was treated with probiotic LC86 at a final concentration of 3×10^7^ CFU/mL. All groups were maintained at 28°C with daily renewal of treatment solutions. At the end of the experiment, cranial bones were evaluated via fluorescence microscopy and quantitative analysis of fluorescence intensity (**Figure 1A**).

DXMS-induced osteogenic injury model in transgenic zebrafish (21, 22): A separate osteogenic injury model was developed using 3 dpf transgenic zebrafish expressing green fluorescence in osteoblasts (Tg (OlaSp7:nlsGFP) cy25) to evaluate the effect of LC86 on osteogenesis. Zebrafish were randomly divided into four groups (CTL, MC, ALN, and LC86), with 30 fish per well in 3 mL of water in 6-well plates. Except for the CTL group, the other groups were treated with 10 μM DXMS for 4 days to induce osteogenic impairment. The ALN group received alendronate sodium (5.00 μg/mL), and the LC86 group received probiotic LC86 at 3×10^7^ CFU/mL. Water and treatments were refreshed daily. At the conclusion of the experiment, zebrafish were imaged using fluorescence microscopy to assess hard bone fluorescence, and pixel intensity values were quantified to evaluate mineralization levels (**Figure 1B**).

**Figure 2:**
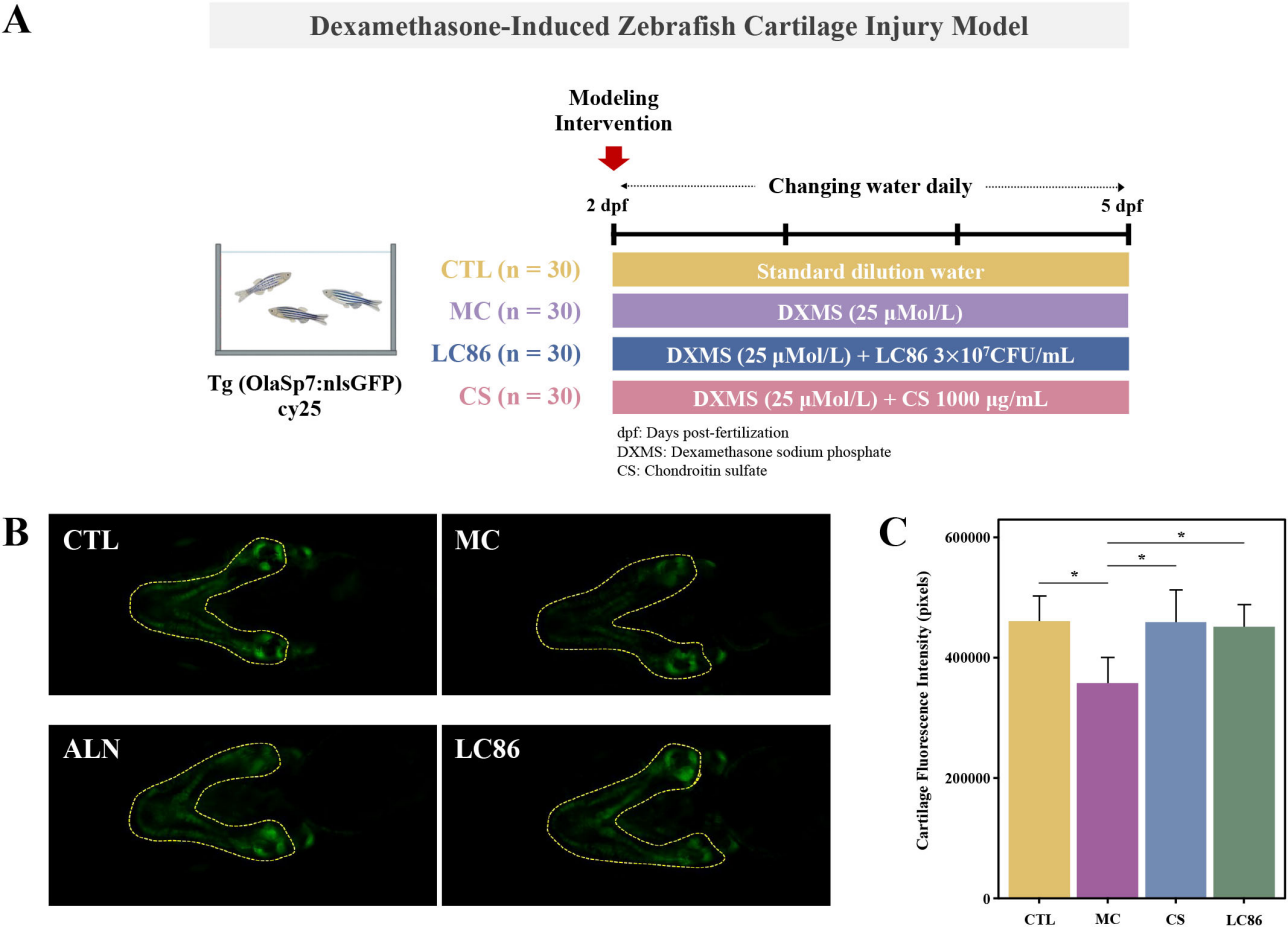
Establishment of a Dexamethasone (DXMS)-induced cartilage damage model in zebrafish and the effects of *Lacticaseibacillus paracasei* LC86 intervention. **(A)** Cartilage damage model using transgenic green fluorescent zebrafish; **(B)** Cartilage fluorescence intensity in zebrafish after LC86 intervention; **(C)** Quantification of cartilage fluorescence intensity. *indicates *p* < 0.05.

DXMS-induced cartilage injury model in transgenic zebrafish (23): To explore the potential protective effects of LC86 against cartilage damage, a cartilage injury model was established using 2 dpf transgenic zebrafish expressing green fluorescence in cartilage tissue. Fish were assigned to four groups: CTL, model control (MC), positive control (CS), and LC86 treatment group. Thirty fish were placed in each well of a 6-well plate containing 3 mL of water. DXMS was added to all groups except CTL at a concentration of 25 μM to induce cartilage injury. The CS group received 1000 μg/mL of chondroitin sulfate as a positive control, and the LC86 group received 3×10^7^ CFU/mL of LC86. All fish were maintained at 28°C with daily solution changes. After 3 days of treatment, zebrafish were collected for imaging, and cartilage integrity was assessed by fluorescence intensity analysis using microscopy (**Figure 2A**).

### Alizarin red staining and fluorescence microscopy imaging

Alizarin red staining and fluorescence microscopy imaging are critical techniques for assessing bone mineralization. Alizarin red dye specifically binds to the calcium salts in bone tissue, making it used for quantifying the degree of bone mineralization (24). At the end of the experiment, 10 zebrafish were randomly selected from each experimental group, placed under a fluorescence microscope for imaging. Images were analyzed using the NIS-Elements D3.20 advanced image processing software to collect data on the fluorescence intensity of the skull, hard bone, and cartilage.

### Detection of bone-related gene expression

At the end of the experiment, three independent biological samples were randomly selected from each group, with each replicate consisting of 30 pooled zebrafish larvae. Total RNA was separately from each biological replicate using an automatic nucleic acid extractor, and its concentration and purity were measured using a UV-Visible spectrophotometer (A_260_/A_280_ ratio). From each sample, 2.00 μg of total RNA was used to synthesize 20.0 μL of cDNA according to the guidelines provided by the cDNA first strand synthesis kit. Subsequently, real-time quantitative PCR (q-PCR) was employed to measure the expression levels of genes associated with bone formation, including *β-actin*, sp*7*, *runx2a*, *bmp2a*, *bmp4*, and *col2a1a*. Throughout this process, *β-actin* served as the internal reference gene, facilitating the calculation of relative RNA expression levels for sp*7*, *runx2a*, *bmp2a*, *bmp4*, and *col2a1a*. The related primer sequences are detailed in **Supplementary Table S1**.

### Untargeted metabolomics

Each treatment group consisted of 18 biological replicates, with 30 zebrafish larvae per replicate (totaling 540 larvae per group). After a 4-day intervention, six biological replicates were randomly selected for untargeted metabolomics. For each replicate, 90 zebrafish larvae (pooled from three wells) were washed with ultrapure water, transferred to 1.5 mL Eppendorf tubes, flash-frozen in liquid nitrogen for 3 min, and stored at −80°C. For metabolite extraction, 25 mg of homogenized zebrafish tissue was mixed with 10 μL of internal standard and 800 μL of cold methanol: acetonitrile: water (2:2:1, v/v/v), followed by homogenization with magnetic beads. Samples were precipitated at −20°C for 2 h, centrifuged at 25,000 g at 4°C for 15 min, and 600 μL of supernatant was freeze-dried. The dried extracts were reconstituted in 600 μL of 50% methanol, vortexed, centrifuged again, and the final supernatant was used for analysis. Chromatographic separation was performed using a Waters BEH C18 column (1.7 μm, 2.1 × 100 mm) with a 15-minute gradient at 0.35 mL/min and 45°C. Mass spectrometry was conducted on a Q Exactive HF system in both positive and negative ion modes (scan range m/z 70–1050), using a Top3 DDA strategy. Data were processed with Compound Discoverer 3.3 and annotated against BMDB, mzCloud, and ChemSpider databases. Peak detection, normalization, and inter-group differential analysis were conducted for metabolic profiling.

### DNA extraction and 16S rRNA gene amplicon sequencing

The intestinal microbiota of zebrafish was analyzed using 6 biological replicates per group (n = 6). Four days post-treatment, the zebrafish samples were washed with ultrapure water at 28°C to remove any residual culture, then quickly transferred to 1.5 mL Eppendorf tubes (30 zebrafish per tube, per biological replicate). After removing all liquid, the samples were flash-frozen in liquid nitrogen for 3 min and stored at -80°C for subsequent 16S rRNA gene sequencing analysis. Genomic DNA extraction was performed using the QIAamp DNA Microbiome Kit (Qiagen, Germany) according to the manufacturer’s protocol, with additional mechanical lysis using bead beating (5.5 m/s for 60 s, MP FastPrep-24). DNA quality and concentration were assessed using a NanoDrop 2000 spectrophotometer (Thermo Fisher Scientific) to measure the A_260_/A_280_ ratio (acceptable range: 1.8-2.0) and a Qubit 3.0 Fluorometer (Thermo Fisher Scientific) for DNA quantification (minimum 30 ng DNA required per sample). Post-PCR, the amplification products were purified using the Agencourt AMPure XP magnetic bead system (Beckman Coulter, USA) and dissolved in Elution Buffer to complete the library construction. The library fragment size and concentration were then assessed using an Agilent 2100 Bioanalyzer. Only libraries that passed quality control proceeded to high-throughput sequencing based on the size of the insert fragments. Sequencing data processing involved adapter sequence trimming with cutadapt v2.6 software and removal of low-quality sequences through a sliding window method to obtain high-quality clean data.

### Bioinformatics analysis of 16S rRNA gene amplicons

Raw sequencing reads were demultiplexed and quality-filtered using cutadapt v2.6 to remove adapter sequences. Low-quality bases were removed using QIIME 2 (DADA2 plugin, default parameters). Reads shorter than 400 bp or containing ambiguous bases (N > 2) were discarded. Paired-end reads were assembled into tags using overlapping relationships, forming the full-length V3–V4 region sequences. The merging criteria were as follows (1): minimum overlap length of 15 bp and (2) a maximum mismatch rate of 0.1 in the overlapping region. The merged tags were clustered into OTUs using USEARCH (v7.0.1090_i86linux32). The process included: (1) OTU clustering at 97% similarity using UPARSE software (version 7.1, http://drive5.com/uparse/) (25), generating representative OTU sequences; (2) chimera removal from OTU representatives using UCHIME (v4.2.40); and (3) OTU abundance profiling by aligning all tags back to representative sequences using the usearch_global method to generate the OTU table for each sample. Taxonomic classification was performed using the RDP Classifier (http://rdp.cme.msu.edu/) against the SILVA 138 database, with a confidence threshold of 70%. Alpha and beta diversity analyses were conducted in QIIME 2 (26), including Shannon and Chao1 indices for species diversity, principal coordinate analysis (PCoA) based on Bray-Curtis dissimilarity, and linear discriminant analysis effect size (LEfSe) for biomarker identification. Based on the OTU results, additional analyses such as intersample species complexity, interspecies differences, correlation analysis, and predictive functional profiling using PICRUSt2 were performed.

### Statistical analysis

The non-parametric Kruskal-Wallis test was used for multi-group comparisons. Differences between two groups were assessed using the non-parametric Mann-Whitney U test. A p-value of less than 0.05 was considered statistically significant. Continuous data were represented as mean ± standard deviation. PCoA based on unweighted UniFrac distances was constructed to visualize gut microbiome community parameters (27). LEfSe was conducted using the microeco package, PICRUSt data were evaluated using the Statistical Analysis of Metagenomic Profiles (STAMP, v 2.1.3), and genus-level abundance differences among the three groups were visualized with the heatmap package (28, 29). Statistical charts and graphs of gut microbiome community parameters were generated using the ggplot2 package in R (30). All statistical analyses were performed using R version 4.2.2.

## Results

### Effect of LC86 intervention against osteoporosis in zebrafish

**Figure 1C** illustrated the fluorescence intensity of zebrafish cranial bones under different treatment conditions and their quantification to assess the impact of various interventions on bone mineralization. Qualitative analysis showed that the CTL group displayed normal baseline fluorescence intensity with no apparent abnormalities. Compared to the CTL group, the MC group exhibited slightly reduced fluorescence intensity, suggesting a potential osteoporotic condition. Relative to the MC group, both the ALN group and LC86 group showed significantly enhanced fluorescence intensity. Further quantitative analysis revealed that, compared to the CTL group, the MC group’s fluorescence pixel values significantly decreased (*p* < 0.001), reflecting reduced levels of bone mineralization consistent with an osteoporotic phenotype. In contrast, cranial bone fluorescence intensity pixel values in both the probiotic LC86 and ALN-treated groups were significantly higher (*p* < 0.01 and *p* < 0.001, respectively) than those in the MC group, consistent with the increase in fluorescence intensity. The effect of LC86 intervention was similar to that of the known bone mineralization promoter, sodium alendronate, effectively enhancing bone mineralization.

### Osteogenic efficacy of LC86 intervention in zebrafish

**Figure 1D** demonstrated the fluorescence microscopy images and their quantitative analysis of zebrafish hard bones under different treatment conditions, assessing LC86’s osteogenic impact. Qualitatively, the CTL group displayed the natural state of untreated hard bones, showing baseline green fluorescence. In contrast, the MC group showed significantly diminished fluorescence, indicating reduced mineralization typical of osteoporosis. Conversely, the LC86 and ALN groups showed markedly enhanced fluorescence, suggesting successful promotion of bone mineralization. Quantitative assessments supported these findings. Compared to CTL group, the fluorescence pixel values in the MC group significantly decreased (*p* < 0.001), reflecting lowered bone mineralization levels. However, both LC86 and ALN treatments significantly increased fluorescence in hard bones (both *p* < 0.01) compared to the MC group, reversing mineralization losses and potentially boosting structural integrity.

### Protective effect of LC86 intervention on zebrafish cartilage injury

The qualitative analysis (**Figure 2B**) revealed that CTL group exhibited baseline fluorescence, indicating healthy cartilage, whereas the MC group’s reduced fluorescence in specific areas suggested potential cartilage damage or decreased mineralization due to the model conditions. Both the ALN and LC86 groups exhibited significant enhancements in fluorescence intensity, especially the ALN group. Quantitative analysis (**Figure 2C**) corroborated the qualitative findings, showing significantly lower fluorescence pixel values (*p* < 0.05) in MC group compared to CTL group, confirming reduced cartilage mineralization. Conversely, the ALN and LC86 groups showed significantly higher fluorescence pixel values (both *p* < 0.05), indicating that these treatments reversed the mineralization loss and potentially enhanced the cartilage’s structural integrity. Although ALN effects were slightly superior, LC86 also demonstrated significant protective benefits.

### Impact of LC86 intervention on gene expression in osteoporotic zebrafish

**Figure 3:**
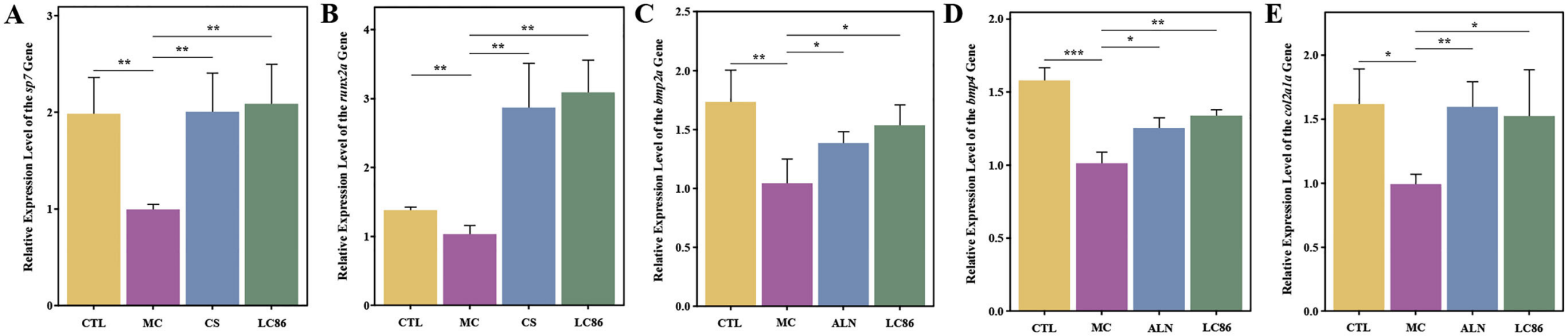
Effects of *Lacticaseibacillus paracasei* LC86 on bone health-related gene expression in the osteoporotic zebrafish model. **(A-E)** Changes in the expression levels of the genes *sp7*, *runx2a*, *bmp2a*, *bmp4*, and *col2a1a* across different groups. *indicates *p* < 0.05, **indicates *p* < 0.01, ***indicates *p* < 0.001.

At the end of study, total RNA was extracted from zebrafish, with UV-visible spectrophotometry confirming high quality suitable for q-PCR (A260/A280 ratios between 1.8-2.2, **Supplementary Table S2**). **Figure 3** analyzed bone health-related gene expression in the zebrafish model to evaluate the effects of LC86. Compared to CTL group, *sp7* gene expression in MC group decreased significantly (*p* < 0.01), but notably recovered in the LC86 and CS groups (both *p* < 0.01), with LC86 showing greater enhancement. RUNX family transcription factor 2a (*runx2a*), essential for bone development, was reduced in MC group (*p* < 0.01), indicating suppressed bone formation. Post-intervention, both LC86 and CS significantly boosted *runx2a* expression, with LC86 performing slightly better. Expression levels of *bmp2a* and *bmp4*, crucial for bone formation and repair, dropped in MC group but were significantly elevated after LC86 and CS treatment, partially reversing osteoporosis effects. Type II collagen (*col2a1a*) expression also increased substantially post-intervention, improving connective tissue integrity, though slightly less effectively with LC86 than CS. Overall, LC86 markedly mitigated gene expression downregulation in the osteoporotic model.

### Untargeted metabolomic analysis of LC86 intervention in osteoporotic zebrafish

**Figure 4:**
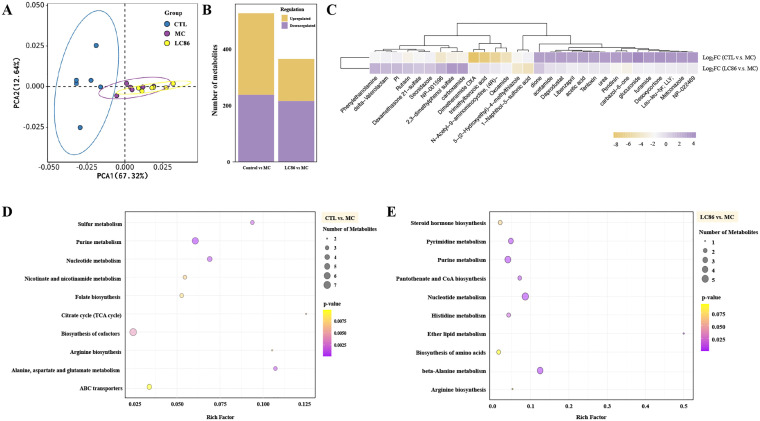
Untargeted metabolomics analysis of *Lacticaseibacillus paracasei* LC86 intervention in the osteoporotic zebrafish model. **(A)** PCA score plot showing overall sample distribution trends (x-axis: PC1, y-axis: PC2, ellipses represent 95% confidence intervals); **(B)** Number and direction of regulation of differential metabolites (x-axis: comparison groups, y-axis: number of differential metabolites; yellow: significantly upregulated, purple: significantly downregulated); **(C)** Log_2_FC values of shared differential metabolites between Control/Model and LC86/Model, showing the top 15 significantly upregulated and downregulated metabolites; **(D, E)** Metabolic pathway enrichment for Control/Model and LC86/Model. The x-axis represents the Rich Factor, quantifying the statistical significance for each pathway. The y-axis lists the metabolic pathways. Bubble size shows the number of differentially expressed metabolites, indicating the pathway’s relative importance between groups. Bubble color depth signifies p-value significance, with darker shades denoting higher statistical significance.

As illustrated in **Figure 4A**, PCA analysis revealed distinct distribution trends between groups, with clear separation between CTL and MC groups indicating significant metabolic differences. Overlaps between LC86 and MC suggest some shared metabolites. **Figure 4B**, through univariate and multivariate analyses, identified 528 differential metabolites between CTL and MC groups, with 290 upregulated and 238 downregulated. In LC86 group compared to MC, 366 differential metabolites were identified, including 150 upregulated and 216 downregulated, suggesting that LC86 intervention partially restored or regulated the metabolic status disrupted by the disease model. Further analysis of differential metabolites between (CTL vs. MC) and (LC86 vs. MC) groups revealed 144 shared metabolites with significant expression trends. Specifically, the top 15 upregulated and downregulated metabolites post-LC86 intervention (**Figure 4C**, **Supplementary Table S3**) reflected significant metabolic activity changes. Upregulated metabolites like Metconazole and Daprodustat, associated with antifungal activity and erythropoiesis, suggest an enhancement or protective effect on metabolism by LC86. Downregulated metabolites like Dimethenamide OXA and Dexamethasone 21-sulfate indicate a potential reduction in metabolites associated with inflammation or chronic stress. Log_2_FC values further quantified these changes, with positive values indicating upregulation and negative values indicating downregulation. By modulating these metabolic pathways, LC86 may exhibit anti-inflammatory, immunomodulatory, and antioxidant effects. Metabolic pathway enrichment analysis based on the KEGG database helped elucidate significant changes in the metabolic pathways, aiding in the interpretation of the biological phenotype. Metabolic pathways with a p-value below 0.05 were defined as significantly enriched, and the top ten pathways with the smallest p-values were plotted as bar graphs. Results in **Figures 4D, E** show that the top three enriched pathways with the most differential metabolites between CTL and MC were purine metabolism, nucleotide metabolism, and alanine, aspartate, and glutamate metabolism, while in LC86 versus MC, the top pathways were nucleotide metabolism, beta-alanine metabolism, and purine metabolism.

### Impact of LC86 intervention on gut microbiome structure and function in osteoporotic zebrafish model

**Figure 5:**
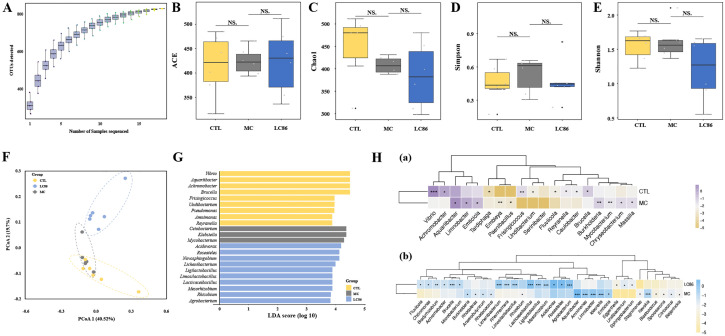
Effects of *Lacticaseibacillus paracasei* LC86 on gut microbiota in an osteoporotic zebrafish model. **(A)** Species accumulation curve; **(B-E)** Alpha diversity analysis showing ACE, Chao1, Simpson, and Shannon indices (NS. indicates *p* > 0.05); **(F)** Unweighted UniFrac principal coordinates analysis (PCoA) showing beta-diversity changes post-LC86 intervention; **(G, H)** Linear Discriminant Analysis Effect Size (LEfSe) and Statistical Analysis of Metagenomic Profiles (STAMP) analyses revealing genus-level changes in relative abundance within the gut microbiota.

Microbial diversity analysis: Species accumulation curves (**Figure 5A**) indicated that sequencing depth was sufficient, with species richness reaching a saturation plateau across all groups. Alpha diversity indices (ACE, Chao1, Simpson, and Shannon; **Figures 5B–E**) showed a trend of reduced microbial diversity in the MC group compared to CTL group, although the differences were not statistically significant. LC86 intervention did not significantly alter these diversity indices. Beta diversity analysis suggested some overlap in species composition between CTL and MC groups, but no distinct clustering was observed post-LC86 intervention, indicating greater sample dispersion. These results suggest that while LC86 did not significantly impact alpha diversity, it modulated the overall structure of the gut microbiome.

Taxonomic composition changes: Using LEfSe and STAMP, significant differences were identified at the genus level between groups. LEfSe analysis (**Figure 5G**) identified significant taxonomic shifts. LEfSe analysis revealed enrichment of pathogenic genera like *Mycobacterium* and *Klebsiella* in MC group, while beneficial genera such as *Ligilactobacillus*, *Limosilactobacillus*, *Lacticaseibacillus*, and *Mesorhizobium* increased in LC86 group, highlighting the beneficial impact of LC86. STAMP analysis further detailed these variations. *Vibrio*, *Achromobacter*, and *Brucella* decreased in the MC group, whereas *Burkholderia*, *Mycobacterium*, and *Aquariibacter* increased (**Figure 5H**). Notably, LC86 reversed these changes, significantly reducing harmful bacteria like *Achromobacter* and *Aquariibacter* and enhancing beneficial ones including *Lacticaseibacillus* and *Ligilactobacillus* (**Figure 5H**).

**Figure 6:**
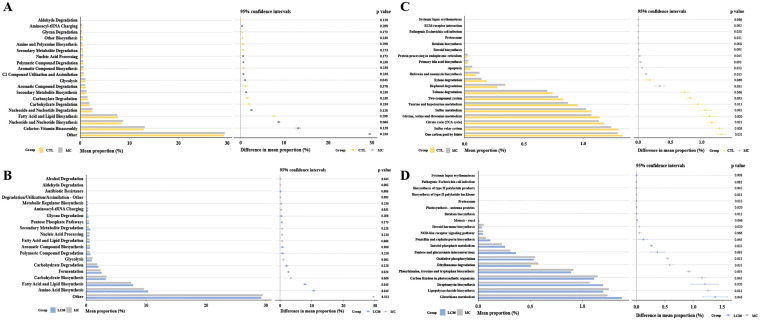
Functional analysis using PICRUSt and STAMP predicted MetaCyc Level 2 and KEGG Level 3 pathways. **(A, B)** MetaCyc Level 2 metabolic pathway analysis for Control vs. Model, and LC86 vs. Model; **(C, D)** KEGG Level 3 functional analysis for Control vs. Model, and LC86 vs. Model. *Lacticaseibacillus paracasei* LC86.

Functional pathway prediction: MetaCyc Level 2 pathway analysis (**Figures 6A, B**) evaluated the intervention’s effects on metabolic pathways. The glycolysis pathway was significantly more active in the MC group (*p* = 0.045), indicating increased energy metabolism demands in osteoporosis. While not statistically significant, slight increases in nucleoside and nucleotide biosynthesis and co-factor biosynthesis pathways were observed in the MC group, reflecting cellular proliferation, repair, and functional demands in the osteoporosis model. Compared to MC group, LC86 group exhibited significantly greater abundance of antibiotic resistance pathways (*p* = 0.005), suggesting that LC86 may boost resistance gene expression in zebrafish gut microbiome, enhancing antibiotic resistance. Additionally, a higher relative abundance of fermentation pathways in LC86 group (*p* = 0.020) implies that LC86 could enhance gut microecology by stimulating fermentation activity. Although differences in the aminoacyl-tRNA charging pathway were subtle (*p* = 0.031), its slightly reduced abundance in LC86 group suggests that LC86 may have optimized protein synthesis efficiency. Additionally, the alcohol degradation pathway was notably lower in LC86 group (*p* = 0.045), indicating a possible decrease in harmful metabolite degradation. Furthermore, the significant increase in fatty acid and lipid biosynthesis pathways in LC86 group (*p* = 0.045) implies that the probiotic could enhance bone health by boosting lipid metabolism.

Analysis of KEGG Level 3 pathways evaluated the impact of LC86 on osteoporotic zebrafish (**Figures 6C, D**). Relative to CTL group, MC group exhibited significant reductions in sulfur transfer, taurine and hypotaurine, glycine, serine and threonine metabolism, and citrate cycle (all *p* < 0.05). Increases were observed in betaine biosynthesis, butaphosphan and neomycin biosynthesis, apoptosis, proteasome activity, and pathways related to pathogenic *E. coli* infection (all *p* < 0.05). Compared to MC, LC86 group showed higher activity in pathways associated with systemic lupus erythematosus, streptomycin biosynthesis, and myo-inositol phosphate metabolism (all *p* < 0.05), and reductions in the proteasome, betaine biosynthesis, lipopolysaccharide biosynthesis, photosynthesis-antenna proteins, NOD-like receptor signaling, oxidative phosphorylation, and styrene degradation (all *p* < 0.05).

## Discussion

This study highlights the potential therapeutic benefits of LC86 in treating osteoporosis and cartilage damage in a zebrafish model, emphasizing the complex relationship between gut microbiome and bone health. Our results demonstrate that LC86 alleviates osteoporosis symptoms by modifying the gut microbiome, engaging fatty acid and lipid biosynthesis pathways, and boosting the expression of key bone mineralization genes such as *runx2a* and *bmp2a*. These findings not only provide molecular-level evidence of probiotics’ role in bone health management but also corroborate recent studies in mammalian models that show the gut microbiome’s influence on bone metabolism (31). Furthermore, other studies have shown that exposure to specific probiotic strains, such as *Bacillus subtilis* and *Lactococcus lactis*, promotes osteoblast formation, matrix growth, and mineralization in zebrafish larvae. Using transgenic zebrafish lines expressing fluorescent reporters for *sp7* and *col10a1a*, these probiotics were found to enhance the expression of bone-development genes including *runx2*, *spp1*, and *col10a1a*, even under BMP inhibition conditions (32). Such findings support the concept that probiotics can actively modulate bone development pathways, offering a promising microbiome-based strategy for skeletal health intervention. Importantly, our data show that LC86 offers comparable or superior outcomes to conventional treatments like ALN and CS, with fewer potential side effects, highlighting its promise as a safe, long-term therapeutic alternative.

In validating the effects of LC86 intervention on osteoporotic and cartilage-damaged zebrafish, Alizarin red staining and fluorescence microscopy of cranial and hard bones clearly showed significant enhancements in fluorescence intensity and pixel values following LC86 treatment, indicating substantial improvements in bone mineralization (33). LC86 demonstrated similar effects to traditional bone mineralization promoters like ALN but possibly involves a broader spectrum of biological processes. Coupled with results from metabolic pathway analysis, the glycolysis pathway was activated in MC group compared to CTL, suggesting that the osteoporotic state may trigger higher metabolic demands. LC86 intervention markedly improved these conditions, suggesting its potential to enhance bone health by modulating energy metabolism, aligning with prior studies (34). Furthermore, LC86 not only improved cartilage fluorescence intensity and structural integrity, likely due to its effects on cartilage cell metabolism and reduced inflammatory activity (35, 36), but also showed advantages over traditional treatments like ALN and CS in bone mineralization and cartilage protection, with fewer side effects and potential for long-term management of skeletal health. This underscores its significance in developing safe and effective long-term treatments.

The analysis of LC86’s modulation of key bone health-related genes highlights its potential mechanisms on gut-bone axis. Gene assays in the osteoporotic zebrafish model revealed that LC86 significantly boosted the expression of crucial bone metabolism genes, such as *sp7*, *runx2a*, *bmp2a*, *bmp4*, and *col2a1a*, which are vital for bone formation, mineralization, and osteoporosis pathogenesis (37). Notably, *sp7* and *runx2a*, essential for osteoblast differentiation and maturation, showed increased expression levels (38–40), suggesting that LC86 could directly enhance bone formation by promoting osteoblast activity. Moreover, *bmp2a* and *bmp4*, which are part of the bone morphogenetic protein family that regulates bone tissue formation and repair, were also upregulated (41, 42). This increase points to LC86’s role in stimulating bone tissue regeneration, with previous studies confirming the importance of *bmp* proteins in bone repair (43). Additionally, the elevated expression of *col2a1a*, critical for cartilage health (37), reflects LC86’s ability to support connective tissue integrity and enhance cartilage health, potentially improving overall skeletal health through the cartilage-bone interaction network (44–46). These findings suggest that LC86 not only improves bone health in osteoporotic conditions but also provides a holistic intervention strategy by targeting key biomarkers in bone and cartilage tissues.

Further untargeted metabolomics analysis has revealed deeper insights into the cellular metabolic mechanisms influenced by LC86 intervention. PCA and differential metabolite analysis identified significant metabolic distinctions between LC86 and MC groups, particularly in key pathways such as purine, nucleotide, and beta-alanine metabolism, which are directly linked to bone health and pathology, particularly in bone formation and cartilage repair (47, 48). Osteoporosis involves an imbalance in bone remodeling, often marked by excessive bone resorption. A notable increase in the glycolysis pathway suggests elevated energy demands, a natural adaptation in osteoporotic conditions and aligns with known metabolic reprogramming in diseases (49). LC86 appears to modify this pathway, potentially optimizing energy metabolism to enhance bone cell survival and function. Furthermore, alterations in nucleotide metabolism may indicate a greater need for cell proliferation and repair (50, 51), essential for restoring bone density and aiding cartilage repair. Increases in nucleotide and cofactor biosynthesis suggest a heightened demand for essential building blocks during cell repair, reflecting bone cells’ response to environmental stress, particularly under osteoporotic conditions (34). The enrichment of beta-alanine metabolism could adjust the body’s alkaline balance, beneficial for bone mineralization, pending further validation (52). Gene expression analysis shows that LC86 potentially modulates key bone formation regulators like *sp7* and *runx2a*, suggesting its direct or indirect impact on osteogenic processes. Together with metabolomics data, LC86’s regulatory actions extend to metabolic pathway modulation, underpinning its multifaceted approach to alleviating osteoporosis symptoms. Additionally, enhanced nucleotide and energy metabolism supports necessary cellular functions for bone regeneration. Although functional prediction analysis revealed an increase in antibiotic resistance-related pathways in the LC86 group, this likely reflects intrinsic characteristics of the probiotic strain rather than acquired or transferable resistance. In accordance with EFSA guidelines, strain LC86 was confirmed to be sensitive to all tested antibiotics, supporting its safety profile and suggesting that the predicted resistance-related pathways are not associated with horizontal gene transfer or antimicrobial risk (53). LC86’s influence on the sulfur transfer system, taurine and hypotaurine metabolism, and the citric acid cycle further underscores its potential in modulating the intracellular milieu, particularly in inflammatory and oxidative conditions (54). This supports LC86’s capability to mitigate inflammation in osteoporosis and cartilage damage models. Overall, LC86 not only addresses direct markers of osteoporosis and cartilage damage but also improves cellular health and function through comprehensive metabolic regulation.

While LC86’s metabolic regulation directly improved bone tissue health, we further investigated its effects on the gut-bone axis through microbiome analysis, focusing on how it alters gut microbiota composition. Alpha diversity analysis showed that LC86 intervention did not significantly alter overall microbial richness or evenness in the osteoporosis model but did adjust the proportions of specific microbial communities. Despite no changes in overall diversity metrics, the composition and functional potential of the microbiota may have shifted. LEfSE and STAMP analyses revealed that LC86 increased the abundance of beneficial genera such as *Lacticaseibacillus* and *Ligilactobacillus*, while reducing potentially pathogenic genera like *Mycobacterium*, potentially reducing both gut and systemic inflammation levels (55). Since inflammation drives bone resorption, modulating gut inflammatory responses with probiotics could lessen inflammation-related bone loss (56). Moreover, changes in pathways related to systemic lupus erythematosus and other autoimmune conditions suggest LC86 might regulate systemic inflammation and immune balance, impacting bone remodeling and cartilage protection (57, 58). Besides, gut microbiota can directly affect bone metabolism via metabolic products like short-chain fatty acids (SCFAs), which are key metabolites from microbial fermentation known to promote bone formation and influence mesenchymal stem cells via G-protein-coupled receptors (11, 59). LC86 could enhance SCFAs production by fostering beneficial gut bacteria, positively influencing bone metabolism. Changes in the gut microbiota also relate closely to the absorption of calcium and other essential minerals for bone health (60). By improving the gut environment and nutrient absorption, probiotics can directly improve bone mineralization and density (60, 61). These insights underscore the broad potential of microbial interventions in bone health management, especially by modifying the gut microbiota to affect host metabolism and immune responses. The integration of metabolomics and microbiome data offers a comprehensive view of how LC86 might mitigate osteoporosis by regulating specific biological pathways and microbial communities.

Although this study highlights the potential of LC86 in promoting bone health, there are several limitations to consider. The zebrafish model, while widely used in bone research due to its genetic tractability and transparency for *in vivo* imaging, differs from mammalian systems in terms of skeletal composition, hormonal regulation, and drug metabolism. These physiological differences may impact the direct translation of findings to human conditions, particularly regarding drug efficacy and dosing strategies. Additionally, *in vivo* zebrafish studies are limited in their ability to dissect complex molecular interactions at the cellular level. Our study primarily focused on gene expression related to bone mineralization, and further research is needed to elucidate the precise molecular mechanisms by which LC86 influences bone metabolism. Future studies should incorporate mammalian models and *in vitro* cellular assays to validate these findings and optimize the clinical relevance of probiotic interventions for bone health.

## Conclusion

In conclusion, this study confirms LC86’s efficacy in a zebrafish model of osteoporosis, notably enhancing bone mineralization and microstructure. LC86 significantly improved both bone tissue health and the gut microbiota structure, while influencing key metabolic pathways including energy, fermentation, and lipid metabolism, highlighting the critical role of the gut-bone axis in bone health. These results support the development of novel bone health treatments based on microbial interventions. Further studies are needed to explore LC86’s clinical application potential.

## Data Availability

The datasets presented in this study can be found in online repositories. The names of the repository/repositories and accession number(s) can be found below: https://www.ncbi.nlm.nih.gov/, PRJNA1134082.

## References

[1] Genant HK Cooper C Poor G Reid I Ehrlich G Kanis J. Interim report and recommendations of the world health organization task-force for osteoporosis. Osteoporosis Int. (1999) 10:259. doi: 10.1007/s001980050224 10692972

[2] Blackie R. Diagnosis, assessment and management of osteoporosis. Prescriber. (2020) 31:14–9. doi: 10.1002/psb.v31.1

[3] Chen C Dong B Wang Y Zhang Q Wang B Feng S. The role of bacillus acidophilus in osteoporosis and its roles in proliferation and differentiation. J Clin Lab Anal. (2020) 34:e23471. doi: 10.1002/jcla.23471 32779308 PMC7676190

[4] Marcacci M Filardo G Kon E. Treatment of cartilage lesions: what works and why? Injury. (2013) 44:S11–S5. doi: 10.1016/S0020-1383(13)70004-4 23351863

[5] Li M Yin H Yan Z Li H Wu J Wang Y. The immune microenvironment in cartilage injury and repair. Acta Biomaterialia. (2022) 140:23–42. doi: 10.1016/j.actbio.2021.12.006 34896634

[6] Iantomasi T Romagnoli C Palmini G Donati S Falsetti I Miglietta F. Oxidative stress and inflammation in osteoporosis: molecular mechanisms involved and the relationship with micrornas. Int J Mol Sci. (2023) 24:3772. doi: 10.3390/ijms24043772 36835184 PMC9963528

[7] Rodan GA Martin TJ. Therapeutic approaches to bone diseases. Science. (2000) 289:1508–14. doi: 10.1126/science.289.5484.1508 10968781

[8] Collins FL Rios-Arce ND Schepper JD Parameswaran N McCabe LR. The potential of probiotics as a therapy for osteoporosis. Microbiol Spectr. (2017) 5. doi: 10.1128/microbiolspec.bad-0015-2016 PMC5710820 28840819

[9] Black DM Rosen CJ. Postmenopausal osteoporosis. New Engl J Med. (2016) 374:254–62. doi: 10.1056/NEJMcp1513724 26789873

[10] Ahlawat S Asha Sharma KK. Gut–organ axis: A microbial outreach and networking. Lett Appl Microbiol. (2021) 72:636–68. doi: 10.1111/lam.13333 32472555

[11] Chen Y-C Greenbaum J Shen H Deng H-W. Association between gut microbiota and bone health: potential mechanisms and prospective. J Clin Endocrinol Metab. (2017) 102:3635–46. doi: 10.1210/jc.2017-00513 PMC5630250 28973392

[12] Zendeboodi F Khorshidian N Mortazavian AM da Cruz AG. Probiotic: conceptualization from a new approach. Curr Opin Food Sci. (2020) 32:103–23. doi: 10.1016/j.cofs.2020.03.009

[13] Lyu Z Hu Y Guo Y Liu D. Modulation of bone remodeling by the gut microbiota: A new therapy for osteoporosis. Bone Res. (2023) 11:31. doi: 10.1038/s41413-023-00264-x 37296111 PMC10256815

[14] Plavnik I Scott M. Effects of additional vitamins, minerals, or brewer's yeast upon leg weaknesses in broiler chickens. Poultry Sci. (1980) 59:459–64. doi: 10.3382/ps.0590459 7413573

[15] Parvaneh K Ebrahimi M Sabran MR Karimi G Hwei ANM Abdul-Majeed S. Probiotics (Bifidobacterium longum) increase bone mass density and upregulate sparc and bmp-2 genes in rats with bone loss resulting from ovariectomy. BioMed Res Int. (2015) 2015:897639. doi: 10.1155/2015/897639 26366421 PMC4558422

[16] Britton RA Irwin R Quach D Schaefer L Zhang J Lee T. Probiotic L. Reuteri treatment prevents bone loss in a menopausal ovariectomized mouse model. J Cell Physiol. (2014) 229:1822–30. doi: 10.1002/jcp.24636 PMC4129456 24677054

[17] Dietrich K Fiedler IA Kurzyukova A López-Delgado AC McGowan LM Geurtzen K. Skeletal biology and disease modeling in zebrafish. J Bone Mineral Res. (2021) 36:436–58. doi: 10.1002/jbmr.4256 33484578

[18] Spoorendonk K Hammond C Huitema L Vanoevelen J Schulte-Merker S. Zebrafish as a unique model system in bone research: the power of genetics and *in vivo* imaging. J Appl Ichthyology. (2010) 26:219–24. doi: 10.1111/j.1439-0426.2010.01409.x

[19] Howe K Clark MD Torroja CF Torrance J Berthelot C Muffato M. The zebrafish reference genome sequence and its relationship to the human genome. Nature. (2013) 496:498–503. doi: 10.1038/nature12111 23594743 PMC3703927

[20] Cai Y Dong Y Han M Jin M Liu H Gai Z. Lacticaseibacillus paracasei lc86 mitigates age-related muscle wasting and cognitive impairment in samp8 mice through gut microbiota modulation and the regulation of serum inflammatory factors. Front Nutr. (2024) 11:1390433. doi: 10.3389/fnut.2024.1390433 38873561 PMC11169942

[21] Luo Q Liu S Xie L Yu Y Zhou L Feng Y. Resveratrol ameliorates glucocorticoid-induced bone damage in a zebrafish model. Front Pharmacol. (2019) 10:195. doi: 10.3389/fphar.2019.00195 30971915 PMC6444061

[22] Jiang RX Hu N Deng YW Hu LW Gu H Luo N. Potential therapeutic role of spermine via rac1 in osteoporosis: insights from zebrafish and mice. Zool Res. (2024) 45:367–80. doi: 10.24272/j.issn.2095-8137.2023.371 PMC11017079 38485506

[23] Jiang Y Xin N Yang J Wu W Wang M Feng N. Prednisolone suppresses collagen-encoding gene expression causing cartilage defects in zebrafish larvae. Environ Toxicol Pharmacol. (2021) 87:103719. doi: 10.1016/j.etap.2021.103719 34332081

[24] Puchtler H Meloan SN Terry MS. On the history and mechanism of alizarin and alizarin red S stains for calcium. J Histochem Cytochem. (1969) 17:110–24. doi: 10.1177/17.2.110 4179464

[25] Edgar RC. Uparse: highly accurate otu sequences from microbial amplicon reads. Nat Methods. (2013) 10:996–8. doi: 10.1038/nmeth.2604 23955772

[26] Caporaso JG Kuczynski J Stombaugh J Bittinger K Bushman FD Costello EK. Qiime allows analysis of high-throughput community sequencing data. Nat Methods. (2010) 7:335–6. doi: 10.1038/nmeth.f.303 PMC3156573 20383131

[27] Oksanen J Blanchet F Friendly M Kindt R Legendre P McGlinn D. Vegan: Community Ecology Package. R Package Version 2.5-6. 2019. Vienna, Austria: The R Foundation for Statistical Computing. (2019).

[28] Liu C Cui Y Li X Yao M. Microeco: an R package for data mining in microbial community ecology. FEMS Microbiol Ecol. (2021) 97:(2). doi: 10.1093/femsec/fiaa255 33332530

[29] Parks DH Tyson GW Hugenholtz P Beiko RG. Stamp: statistical analysis of taxonomic and functional profiles. Bioinformatics. (2014) 30:3123–4. doi: 10.1093/bioinformatics/btu494 PMC4609014 25061070

[30] Gómez-Rubio V. Ggplot2 - elegant graphics for data analysis (2nd edition). J Stat Software. (2017) 77:1–3. doi: 10.18637/jss.v077.b02

[31] Villa CR Ward WE Comelli EM. Gut microbiota-bone axis. Crit Rev Food Sci Nutr. (2017) 57:1664–72. doi: 10.1080/10408398.2015.1010034 26462599

[32] Sojan JM Raman R Muller M Carnevali O Renn J. Probiotics enhance bone growth and rescue bmp inhibition: new transgenic zebrafish lines to study bone health. Int J Mol Sci. (2022) 23:4748. doi: 10.3390/ijms23094748 35563140 PMC9102566

[33] Arnold A Dennison E Kovacs CS Mannstadt M Rizzoli R Brandi ML. Hormonal regulation of biomineralization. Nat Rev Endocrinol. (2021) 17:261–75. doi: 10.1038/s41574-021-00477-2 33727709

[34] Lee W-C Guntur AR Long F Rosen CJ. Energy metabolism of the osteoblast: implications for osteoporosis. Endocrine Rev. (2017) 38:255–66. doi: 10.1210/er.2017-00064 PMC5460680 28472361

[35] Korotkyi O Huet A Dvorshchenko K Kobyliak N Falalyeyeva T Ostapchenko L. Probiotic composition and chondroitin sulfate regulate tlr-2/4-mediated nf-Kb inflammatory pathway and cartilage metabolism in experimental osteoarthritis. Probiotics Antimicrobial Proteins. (2021) 13:1018–32. doi: 10.1007/s12602-020-09735-7 33459997

[36] Cho K-H Na HS Jhun J Woo JS Lee AR Lee SY. Lactobacillus (La-1) and butyrate inhibit osteoarthritis by controlling autophagy and inflammatory cell death of chondrocytes. Front Immunol. (2022) 13:930511. doi: 10.3389/fimmu.2022.930511 36325344 PMC9619036

[37] Raman R Antony M Nivelle R Lavergne A Zappia J Guerrero-Limón G. The osteoblast transcriptome in developing zebrafish reveals key roles for extracellular matrix proteins col10a1a and fbln1 in skeletal development and homeostasis. Biomolecules. (2024) 14:139. doi: 10.3390/biom14020139 38397376 PMC10886564

[38] Zhang Y Gao Y Cai L Li F Lou Y Xu N. Microrna-221 is involved in the regulation of osteoporosis through regulates runx2 protein expression and osteoblast differentiation. Am J Trans Res. (2017) 9:126. PMC5250709 28123639

[39] Wang JS Tokavanich N Wein MN. Sp7: from bone development to skeletal disease. Curr osteoporosis Rep. (2023) 21:241–52. doi: 10.1007/s11914-023-00778-7 PMC10758296 36881265

[40] Valenti MT Marchetto G Mottes M Dalle Carbonare L. Zebrafish: A suitable tool for the study of cell signaling in bone. Cells. (2020) 9:1911. doi: 10.3390/cells9081911 32824602 PMC7465296

[41] Chan WCW Tan Z To MKT Chan D. Regulation and role of transcription factors in osteogenesis. Int J Mol Sci. (2021) 22:5445. doi: 10.3390/ijms22115445 34064134 PMC8196788

[42] Håkelien A-M Bryne JC Harstad KG Lorenz S Paulsen J Sun J. The regulatory landscape of osteogenic differentiation. Stem Cells. (2014) 32:2780–93. doi: 10.1002/stem.1759 24898411

[43] Segredo-Morales E Garcia-Garcia P Evora C Delgado A. Bmp delivery systems for bone regeneration: healthy vs osteoporotic population. Review. J Drug delivery Sci Technol. (2017) 42:107–18. doi: 10.1016/j.jddst.2017.05.014

[44] Korotkyi O Kyriachenko Y Kobyliak N Falalyeyeva T Ostapchenko L. Crosstalk between gut microbiota and osteoarthritis: A critical view. J Funct Foods. (2020) 68:103904. doi: 10.1016/j.jff.2020.103904

[45] Zuo G Zhuang P Yang X Jia Q Cai Z Qi J. Regulating chondro-bone metabolism for treatment of osteoarthritis via high-permeability micro/nano hydrogel microspheres. Advanced Sci. (2024) 11:2305023. doi: 10.1002/advs.202305023 PMC10837371 38084002

[46] Paul AK Paul A Jahan R Jannat K Bondhon TA Hasan A. Probiotics and amelioration of rheumatoid arthritis: significant roles of lactobacillus casei and lactobacillus acidophilus. Microorganisms. (2021) 9:1070. doi: 10.3390/microorganisms9051070 34065638 PMC8157104

[47] Yang K Li J Tao L. Purine metabolism in the development of osteoporosis. Biomedicine Pharmacotherapy. (2022) 155:113784. doi: 10.1016/j.biopha.2022.113784 36271563

[48] Agrawal A Jørgensen NR. Extracellular purines and bone homeostasis. Biochem Pharmacol. (2021) 187:114425. doi: 10.1016/j.bcp.2021.114425 33482152

[49] Li D Gao Z Li Q Liu X Liu H. Cuproptosis-a potential target for the treatment of osteoporosis. Front Endocrinol. (2023) 14:1135181. doi: 10.3389/fendo.2023.1135181 PMC10196240 37214253

[50] Devignes C-S Carmeliet G Stegen S. Amino acid metabolism in skeletal cells. Bone Rep. (2022) 17:101620. doi: 10.1016/j.bonr.2022.101620 36120644 PMC9475269

[51] Yang J Ueharu H Mishina Y. Energy metabolism: A newly emerging target of bmp signaling in bone homeostasis. Bone. (2020) 138:115467. doi: 10.1016/j.bone.2020.115467 32512164 PMC7423769

[52] Hou J-L Yang W-Y Zhang Q Feng H Wang X-B Li H. Integration of metabolomics and transcriptomics to reveal the metabolic characteristics of exercise-improved bone mass. Nutrients. (2023) 15:1694. doi: 10.3390/nu15071694 37049535 PMC10097349

[53] Chen T Zhao Y Fan Y Dong Y Gai Z. Genome sequence and evaluation of safety and probiotic potential of lacticaseibacillus paracasei lc86 and lacticaseibacillus casei lc89. Front Microbiol. (2025) 15:1501502. doi: 10.3389/fmicb.2024.1501502 39931277 PMC11808145

[54] Olechnowicz J Tinkov A Skalny A Suliburska J. Zinc status is associated with inflammation, oxidative stress, lipid, and glucose metabolism. J Physiol Sci. (2018) 68:19–31. doi: 10.1007/s12576-017-0571-7 28965330 PMC5754376

[55] Yao M Lu Y Zhang T Xie J Han S Zhang S. Improved functionality of ligilactobacillus salivarius li01 in alleviating colonic inflammation by layer-by-layer microencapsulation. NPJ Biofilms Microbiomes. (2021) 7:58. doi: 10.1038/s41522-021-00228-1 34244520 PMC8270932

[56] Ke K Arra M Abu-Amer Y. Mechanisms underlying bone loss associated with gut inflammation. Int J Mol Sci. (2019) 20:6323. doi: 10.3390/ijms20246323 31847438 PMC6940820

[57] Panopalis P Yazdany J. Bone health in systemic lupus erythematosus. Curr Rheumatol Rep. (2009) 11:177–84. doi: 10.1007/s11926-009-0024-2 19604461

[58] Zhu L Hua F Ding W Ding K Zhang Y Xu C. The correlation between the th17/treg cell balance and bone health. Immun Ageing. (2020) 17:30. doi: 10.1186/s12979-020-00202-z 33072163 PMC7557094

[59] Lucas S Omata Y Hofmann J Böttcher M Iljazovic A Sarter K. Short-chain fatty acids regulate systemic bone mass and protect from pathological bone loss. Nat Commun. (2018) 9:55. doi: 10.1038/s41467-017-02490-4 29302038 PMC5754356

[60] Dubey MR Patel VP. Probiotics: A promising tool for calcium absorption. Open Nutr J. (2018) 12. doi: 10.2174/1874288201812010059

[61] Weaver CM. Diet, gut microbiome, and bone health. Curr osteoporosis Rep. (2015) 13:125–30. doi: 10.1007/s11914-015-0257-0 PMC4996260 25616772

